# Cutaneous cryptococcosis simulating pyoderma gangrenosum

**DOI:** 10.1590/0037-8682-0120-2020

**Published:** 2020-06-12

**Authors:** Agatha Ramos Oppenheimer, Neusa Yuriko Sakai Valente, Diego Henrique Morais Silva

**Affiliations:** 1 Hospital do Servidor Público Estadual de São Paulo, Programa de Residência Médica em Dermatologia, São Paulo, SP, Brasil.; 2 Hospital do Servidor Público Estadual de São Paulo, Departamento de Dermatologia, São Paulo, SP, Brasil.

A 53-year-old man presented with a 4-month history of painful lesions on the inner side of his left thigh with drainage of purulent secretion. His past medical history included gouty arthritis and chronic use of corticosteroids for eight years. On physical examination, he had ulcers with high, well-defined edges, covered with necrotic and purulent material on the inner side of the left thigh, resembling pyoderma gangrenosum (Figure A). Histopathological examination revealed dermis with a diffuse infiltrate mixed with lymphocytes, histiocytes, neutrophils and plasma cells. Numerous fungal structures involving yeast in the dermis were observed by the Grocott methanamine silver stain (Figure B). These findings allowed the diagnosis of cutaneous cryptococcosis. Extracutaneous involvement was ruled out by analyzing cerebrospinal fluid, and by performing chest radiography and bronchoalveolar lavage. However, blood cultures exhibited fungal growth. Treatment with 200 mg intravenous fluconazole 12/12 h was instituted, with clinical improvement after the third week, at which point the patient was discharged. After 80 days of oral treatment, the lesions were significantly improved, with approximately 75% regression (Figure C). 

**FIGURE A:**
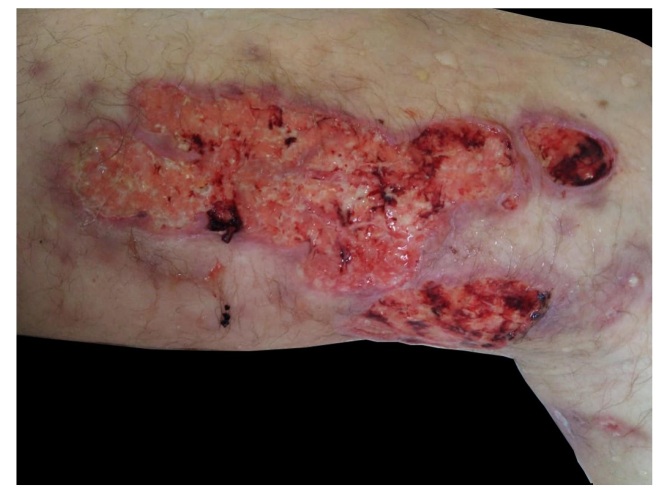
Ulcers with high, well-defined edges, covered with necrotic and purulent material on the inner side of the left thigh.

**FIGURE B:**
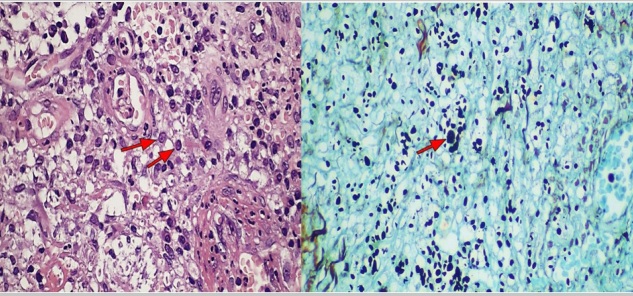
Dermal infiltrate with a predominance of histiocytes containing rounded structures in their cytoplasm, better seen with Grocott (H&E stain, original magnification × 400).

**FIGURE C:**
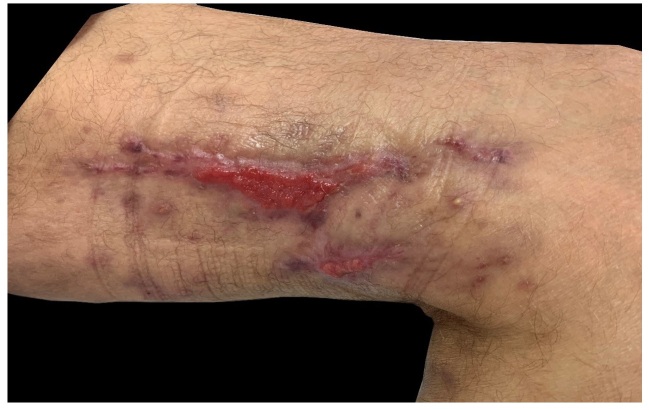
Ulcers on the inner side of the left thigh after 80 days of oral treatment with fluconazole.

Cutaneous cryptococcosis is a rare disease characterized by varied lesion morphology, including umbilicated papules, plaques, nodules, pustules, granulomas, abscesses or ulcerations^1^ ^,^ ^2^. There are few reports in the literature of lesions mimicking pyoderma gangrenosum, which presents ulcers with high and well-defined edges^3^. Cryptococcosis should be considered in the differential diagnosis of refractory ulcers, especially in immunosuppressed patients.
